# Awareness, Treatment, Control of Diabetes Mellitus and the Risk Factors: Survey Results from Northeast China

**DOI:** 10.1371/journal.pone.0103594

**Published:** 2014-07-28

**Authors:** Chang Wang, Yaqin Yu, Xiangyang Zhang, Yong Li, Changgui Kou, Bo Li, Yuchun Tao, Qing Zhen, Huan He, Joseph Sam Kanu, Xufeng Huang, Mei Han, Yawen Liu

**Affiliations:** 1 Department of Epidemiology and Biostatistics, School of Public Health, Jilin University, Changchun City, Jilin Province, P.R. China; 2 Beijing Hui-Long-Guan hospital, Peking University, Beijing, P.R. China; 3 Illawarra Health and Medical Research Institute, University of Wollongong, Wollongong, Australia; Weill Cornell Medical College Qatar, Qatar

## Abstract

**Background:**

The awareness, treatment and control of diabetes mellitus (DM) can effectively reflect on the social status of diabetes conditions. Although several researchers have investigated the awareness, treatment and control rates of diabetes mellitus in China, little is known about their association with risk factors. This study aims to examine the relationship between risk factors and awareness, treatment and control of diabetes mellitus in northeast China.

**Methods:**

A cross-sectional survey was conducted in 2012. Multistage stratified random cluster sampling design was used to select participants aged 18 to 79 years old. The analysis was based on a representative sample of 1,854 adult subjects. Multivariable logistic regression analysis was used to examine socio-demographic factors associated with the levels of awareness, treatment and control of diabetes mellitus.

**Results:**

The awareness, treatment, and control rates of diabetes mellitus were 64.1%, 52.9% and 44.2%, respectively. In the multivariable logistic regression analysis, family history of diabetes was significantly positively associated with awareness (OR, 2.145; 95% CI, 1.600–2.875) and treatment (OR, 2.021; 95% CI, 1.559–2.619) of diabetes mellitus, while negatively associated with control (OR, 0.671; 95% CI, 0.529–0.951). Cigarette smokers and alcohol drinkers were less likely than non-smokers and non-drinkers to be aware of their blood glucose levels (OR, 0.895, 0.614; 95% CI, 0.659–1.216, 0.446–0.844, respectively). Participants who frequently exercise were more likely to be aware of their diabetic conditions than people who never or rarely exercise (OR, 2.003; 95% CI, 1.513–2.651).

**Conclusions:**

We found that the awareness and treatment of diabetes mellitus were positively associated with age and were high in participants with a family history of diabetes and those who exercise frequently, but low for cigarette smokers and alcohol drinkers. Participants with a family history of diabetes had their diabetic condition poorly controlled.

## Introduction

With rapid economic development, population ageing and urbanization, DM has become an important medical problem in nearly all countries [1], [2], [3]. It is estimated that the number of people with DM is expected to rise to 552 million by 2030, with a 69% increase from 2010 to 2030 for developing countries, compared to 20% for developed countries [4], [5]. DM has become a heavy economic burden for every country.

China’s prevalence of DM has been ranked second in the world, and has the largest absolute disease burden that is due to increasing prevalence and low awareness, treatment and control of DM [1], [6]. It is estimated that the age-standardized prevalence of total DM in China is 9.7% (10.6% among men and 8.8% among women) [7]. Early detection of high blood glucose levels and a comprehensive care of diabetes are effective for improving clinical outcomes and quality of life for DM. Therefore, China critically needs government-initiated primary prevention measures for DM, which focuses on the risk factors of the awareness, treatment and control.

The awareness, treatment and control of DM can effectively reflect on the social status of diabetes conditions. Although several researchers have investigated the awareness, treatment and control rates of DM in China [8], [9], little is known about their association with risk factors.

Jilin province is located in northeast China and has a population of about 27 million people. Jilin Department of Health and Jilin University jointly investigated the prevalence of DM and risk factors for awareness, treatment and control of DM in Jilin province in 2012. We used data from this investigation to examine the distribution of awareness, treatment, and control of DM among adult residents in northeast China, as well as how risk factors relate to the awareness, treatment and control of DM.

## Materials and Methods

### Study population

We carried out a large-scale community-based cross-sectional health interview and examination survey on permanent residents aged 18 to 79 years old who had lived in Jilin province for more than six months. The survey respondents were asked to answer each question face-to-face with investigators who had been uniformly trained.

### Sampling method

Five-stage stratified random cluster sampling was used to select the study sample. In the first stage, 32 districts/counties were identified in proportion to population, geographic location and ethnicity, form 9 cities (Changchun, Jilin, Siping, Liaoyuan, Tonghua, Baishan, Songyuan, Baicheng and Yanbian). At the second stage, three or four towns (depending on the size of the district) were selected by stratified random sampling to guarantee the representativeness of each sample. In the third stage, three neighborhood committees were chosen by stratified random sampling from each of the towns previously selected. In the fourth stage, one village from each chosen neighborhood committee was selected by simple random sampling. In the final stage, cluster random sampling was used to identify individuals aged 18 to 79 years old from each of the villages selected for the study.

### Data collection and measurement

A strict quality control system was implemented at each stage of the data collection to ensure uniformity and accuracy of the data. Before the formal investigation, a pre-investigation was conducted to explore the design of the questionnaire. In addition, systematic training for all investigators was organized to teach them on how to administer the screening questionnaire, and how to take anthropometric measurements. Before the interview, the identity of each participant was confirmed by the investigator. The validity of each answered questionnaire was examined by the interviewer after the participant had completed the questionnaire in order to ascertain whether the responses were consistent with the real situation. After the fieldwork, all data were processed by parallel double entry. Three verifications were carried out to check for incomplete or inconsistent responses, and then deleted the missing data that cannot be repaired.

The data collection included an intensive investigation and household survey. The purpose of the investigation was explained to the participants before the interview, and then given the option to sign an informed consent form, and only those who consented to participate in the study proceeded to answering the questions and doing the further investigations. The study was approved by the Institutional Review Board of the School of Public Health, Jilin University, Jilin, China. The demographic information collected included gender, age, family income, level of education, occupation, smoking, drinking, exercise, diagnosis and treatment of diabetes, and self-reported family history of diabetes. In addition, anthropometric measurements including height, weight, and fasting blood sugar were taken.

During the interview, weight and height were determined though standardized protocol and measured in light indoor clothing without shoes. Body mass index (BMI) was calculated as weight in kilograms divided by height in meters squared (kg/m^2^). Fasting plasma glucose (FPG) levels were measured using the Bayer Bai Ankang fingertip blood glucose monitor machine by taking a small drop of blood from a finger onto a strip of paper in the morning after participants fasted for 10 or more hours overnight.

### Definitions

DM was defined as a FPG ≥7.0 mmol/L or self-reported use of anti-diabetic medications during the 2 weeks prior to the examination. Diagnosed diabetes was defined as those meeting the standards for DM [10] or participants reported he/she had previous diagnosis of diabetes by a medical doctor in a hospital above the county level. Individuals with diabetes were considered to be aware of diabetes if they reported having a previous diagnosis of diabetes by medical doctors. Individuals with diabetes were classified as being taking treatment for diabetes if they reported using anti-diabetic medications during the 2 weeks prior to the examination. Among treated diabetes, diabetes was considered to be controlled if FPG≤ 7.0 mmol/L.

Education was divided into three (3) levels: junior school (including those who never attended school, those who attained elementary schooling only, and those who attained junior school); high school (including those who attained high school and a 3- year secondary vocational schooling); and tertiary level of education (including undergraduate and graduate levels of education). Manual labor occupation included production workers, farmers and service workers. Mental labor included office and other technical staffs. Other occupations included students, unemployed and retirees. Body Mass Index (BMI) was classified as: underweight, BMI<18.5 kg/m^2^; normal weight, 18.5≤BMI<24 kg/m^2^; overweight, 24≤BMI<28 kg/m^2^; and obese, BMI≥28 kg/m^2^ [11]. Smoker was defined as a person who smoked more than one cigarette per day in the past 30 days. Drinker was defined as a person who consumed an average of more than one alcoholic drink per week, whether spirits, beer, wine or other forms of alcohol. Participants who were classified as “sometimes exercise” were defined as persons who exercised one or two times a week; those who exercised more than three times a week were defined as “exercise frequently” ; while those don’t or seldom exercise were defined as “never exercise” or “rarely exercise”.

### Statistical analysis

All statistical analyses were weighted to represent the total adult population of Jilin province. The sampling weights were obtained from the 2010 China population census data, and were calculated based on four factors: regional, urban/rural, gender, and age in Jilin province. Frequency distributions were used to describe the awareness, treatment, and control with the categories of participants’ characteristics. The Rao-Scott Chi-square tests corrected for complex sampling design were used to compare the awareness, treatment and control of diabetes in the different groups. Multivariable logistic regressions were used to examine the socio-demographic factors associated with the levels of diabetes awareness, treatment and control, and the odds-ratio (OR) and 95% confidence intervals (CI) were calculated. Sampling errors were estimated by using the primary sampling units and strata provided in the data set. The standard errors of these estimates were corrected for the clustering used in the five-stage stratified random clustering sampling procedure. Data were analyzed using the complex samples function of SPSS version 21.0 and *P*≤0.05 was considered to be statistically significant.

## Results

Of the 18260 participants, a total of 1854 (8.2% after weighted) were diagnosed with DM. The analyzed sample included 1,854 participants, which consisted of 941 women and 913 men. As shown in Table 1, participants aged 55–64 years were the largest age group (29.9%), 63.6% only attained junior school education, 26.1% had a high school education, and 34.0% had a family monthly income of 1000–2000 Yuan. Table 1 also shows that manual labor had a higher percentage than mental labor (46.1% vs. 16.3%); 29.0% had a family history of diabetes; and 30.7% were cigarette smokers at the time of the study; 31.6% were alcohol drinkers.

**Table 1:** Demographic characteristics between different socio-demographic factors.

Category	Subcategory	Frequency (n = 1854)	Percentage (%)	Estimated population
Gender	Female	941	43.4	653
	Male	913	56.6	850
Age (years)	18∼34	50	7.5	113
	35∼44	200	15.0	226
	45∼54	565	28.7	430
	55∼64	681	29.9	448
	65∼79	358	18.9	284
Education	<Junior school	1221	62.6	940
	High school	452	26.1	392
	Undergraduate	181	11.3	170
Income (Chinese Yuan)	<500	463	21.3	321
	500∼	378	19.5	292
	1000∼	610	34.0	511
	2000∼	260	16.1	242
	3000∼	143	9.1	137
Occupation	Manual labor	883	46.1	693
	Mental labor	260	16.3	244
	Other	711	37.6	565
Family history	No	1330	71.0	1066
	Yes	524	29.0	436
Region	Rural	923	44.4	667
	Urban	931	55.6	835
BMI*	Normal	546	29.1	432
	Underweight	28	1.4	21
	Overweight	816	44.8	665
	Obesity	446	24.8	368
Smoker	Never	1082	55.9	840
	Now	533	30.7	461
	Once	239	13.4	202
Drinker	No	1326	68.4	1028
	Yes	528	31.6	475
Exercise	Never or rare	635	34.3	515
	Sometimes	332	19.3	290
	Frequently	887	46.4	697

*BMI, Body mass index. Percentage did not add up to 100% due to missing data.

Table 2 shows the awareness, treatment and control (among treated) rates of DM in relation to the various socio-demographic factors. The awareness, treatment and control percentages of DM were 64.1%, 52.9% and 44.2% respectively. Women had a greater awareness (74.2% vs. 56.4%) and treatment (62.2% vs. 45.7%) of DM than men. The awareness, treatment and control rates increased with advancing age (*P*<0.05). Levels of educations were not significantly associated with control of DM (*P*>0.05). Manual laborers had greater awareness (58.9% vs. 54.8%) and treatment (47.6% vs. 41.2%) of DM compared to mental workers. People with a family history of diabetes (73.0% vs. 60.5%, *P*<0.001) were more likely to be aware of their diabetic conditions and to be treated with anti-diabetic medications than those without the family history (61.4% vs. 49.4%, *P*<0.001), but the percentage of control was significantly higher in those having no family history of diabetes compared to those having family history (47.6% vs. 37.5%, *P* = 0.005). There were no statistically significant associations between BMI and awareness, treatment or control of DM.

**Table 2:** Comparison of awareness, treatment and control of diabetes between various factors [n (%)].

Category	Subcategory	Awareness		Treatment		Control among treated	
		%(n)	Estimated population	%(n)	Estimated population	%(n)	Estimated population
Total		64.1(1245)	963	52.9(1056)	794	44.2(466)	351
Gender	Female	74.2(696)	484	62.2(596)	406	45.8(277)	186
	Male	56.4(549)	479	45.7(460)	388	42.5(189)	165
	*P* value	<0.001		<0.001		0.338	
Age (years)	18∼34	27.4(13)	31	4.7(3)	5	10.4(1)	1
	35∼44	48.8(100)	110	39.4(81)	89	35.8(30)	32
	45∼54	62.7(359)	270	53.5(305)	230	41.2(120)	95
	55∼65	73.5(500)	330	63.2(432)	283	43.1(190)	122
	65∼79	78.3(273)	222	65.6(235)	186	54.6(125)	102
	*P* value	<0.001		<0.001		0.006	
Education	<Junior school	67.5(841)	634	55.7(714)	524	43.9(312)	230
	High school	59.4(293)	233	48.4(250)	190	44.8(113)	85
	Undergraduate	56.6(111)	96	47.1(92)	80	44.4(41)	36
	*P* value	0.011		0.035		0.975	
Income (Chinese Yuan)	<500	70.2(341)	225	59.7(290)	191	39.4(112)	75
	500∼	64.7(242)	189	50.0(197)	146	43.1(86)	63
	1000∼	62.3(398)	319	51.8(341)	265	47.1(163)	125
	2000∼	66.4(183)	161	54.7(156)	132	43.3(70)	57
	3000∼	51.3(81)	70	43.5(72)	59	51.3(35)	31
	*P* value	0.017		0.042		0.374	
Occupation	Manual labor	58.9(552)	408	47.6(461)	330	38.1(180)	126
	Mental labor	54.8(155)	134	41.2(121)	101	42.9(51)	43
	Other	74.6(538)	422	64.3(474)	363	50.1(235)	182
	*P* value	<0.001		<0.001		0.005	
Family history	No	60.5(836)	645	49.4(704)	526	47.6(331)	251
	Yes	73.0(409)	318	61.4(352)	268	37.5(135)	100
	*P* value	<0.001		<0.001		0.005	
Region	Rural	65.8(636)	439	52.7(533)	352	41.1(221)	145
	Urban	62.8(609)	524	53.0(523)	442	46.6(245)	206
	*P* value	0.300		0.929		0.105	
BMI	Normal	62.8(364)	271	50.4(305)	218	44.8(136)	98
	Underweight	53.6(17)	11	41.8(14)	9	23.5(3)	2
	Overweight	64.1(543)	427	54.0(468)	359	43.7(203)	157
	Obesity	66.5(309)	245	55.2(260)	203	45.3(121)	92
	*P* value	0.621		0.420		0.570	
Smoker	Never	68.2(769)	573	56.0(652)	470	46.0(304)	216
	Now	52.8(300)	243	44.6(262)	205	39.1(101)	80
	Once	73.1(176)	147	58.7(142)	118	46.0(61)	54
	*P* value	<0.001		<0.001		0.228	
Drinker	No	70.9(968)	728	59.3(833)	610	45.5(380)	277
	Yes	49.5(277)	235	38.9(223)	185	40.0(86)	74
	*P* value	<0.001		<0.001		0.186	
Exercise	Never or rare	53.4(368)	275	43.9(310)	226	42.2(131)	95
	Sometimes	58.2(207)	169	46.8(176)	136	43.1(77)	59
	Frequently	74.6(670)	520	62.0(570)	432	45.6(258)	197
	*P* value	<0.001		<0.001		0.660	

Data are presented as % (n).

Table 3 shows the results of multivariate logistic regressions for the awareness, treatment and control rates of DM. These were performed to determine whether the statistically significant factors found in the univariate analysis were associated with awareness, treatment and control.

**Table 3:** Multivariate logistic regression for awareness, treatment and control of diabetes mellitus.

Category	Subcategory	Awareness		Treatment		Control among treated	
		OR(95.0% CI)	*P*	OR(95.0% CI)	*P*	OR(95.0% CI)	*P*
Gender	Male v. female	0.640(0.464–0.884)	0.007	0.764(0.572–1.020)	0.068	–	–
Age	35∼44 v. 18∼34	2.611(1.116–6.108)	<0.001	15.789(2.956–84.347)	<0.001	3.949(0.319–48.895)	0.172
	45∼54 v. 18∼34	4.328(1.901–9.854)		27.302(5.269–141.477)		4.799(0.401–57.375)	–
	55∼64 v. 18∼34	5.966(2.597–13.703)		33.474(6.396–175.198)		4.726(0.395–56.494)	–
	65∼79 v. 18∼34	6.951(2.902–16.653)		33.108(6.279–174.583)		6.766(0.558–82.023)	–
Education	High school v. <Junior school	0.948(0.684–1.313)	0.889	1.014(0.755–1.362)	0.778	–	–
	Undergraduate v. <Junior school	0.884(0.521–1.500)		1.196(0.715–2.003)		–	–
Income	500∼ v. <500	0.876(0.604–1.272)	0.049	0.731(0.530–1.007)	0.091	–	–
	1000∼ v. <500	0.737(0.530–1.026)		0.793(0.584–1.079)		–	–
	2000∼ v. <500	1.278(0.819–1.994)		1.178(0.780–1.780)		–	–
	3000∼ v. <500	0.740(0.436–1.255)		0.864(0.509–1.469)		–	–
Occupation	Mental labor v. manual labor	0.989 (0.630–1.550)	0.747	0.789 (0.517–1.181)	0.043	1.228 (0.783–1.927)	0.096
	other v. manual labor	1.212 (0.824–1.526)		1.283 (0.986–1.670)		1.403 (1.030–1.910)	
Family history	Yes v. no	2.145(1.600–2.875)	<0.001	2.021(1.559–2.619)	<0.001	0.710 (0.529–0.951)	0.022
Smoker	Now v. never	0.895(0.659–1.216)	0.007	1.088(0.817–1.450)	0.217	–	–
	Once v. never	1.699(1.147–2.517)		1.373(0.962–1.985)		–	–
Drinker	Yes v. no	0.614(0.446–0.844)	0.003	0.551(0.409–0.742)	<0.001	–	–
Exercise	Sometimes v. never or rare	1.377(0.952–1.991)	<0.001	1.259(0.905–1.752)	0.003	–	–
	Frequently v. never or rare	2.003(1.513–2.651)		1.560(1.209–2.014)		–	–

Results are expressed as Odd Ratio and (95% confidence interval).

–, not retained in the model.

After controlling for potential confounding factors of socio-demographic variables, the analyses showed that older individuals have greater probability of being aware of their diabetic conditions and seek medical treatment than younger individuals, and this is presented in Table 3. A family history of diabetes was significantly associated with awareness (OR, 2.145; 95% CI, 1.600–2.875) and treatment (OR, 2.021; 95% CI, 1.559–2.620) of DM, and participants with a family history of diabetes were less likely than those without to have their diabetes controlled (OR, 0.671; 95% CI, 0.529–0.951). Cigarette smokers and alcohol drinkers were less likely than non-smokers and non-drinkers to be aware of their blood glucose levels (OR, 0.895, 0.614; 95% CI, 0.659–1.216, 0.446–0.844, respectively). Participants who exercise frequently were more likely than those who never or rarely exercise to be aware of their diabetes (OR, 2.003; 95% CI, 1.513–2.651) and take anti-diabetic medication (OR, 1.560; 95% CI, 1.209–2.014).

## Discussion

In recent years, there have been efforts to perform epidemiological studies on diabetes, but little data covers the awareness, treatment, and control of DM in China. Knowing the factors associated with awareness, treatment and control of DM can be very useful in reducing the prevalence of this disease in China. To our knowledge, this is the first large representative population-based survey to provide insightful data on awareness, treatment, and control of DM and associated factors in Jilin province in northeast China.

In our study, we found that the awareness and treatment of DM in Jilin province is higher than that reported in a prior study in China [6], but much lower than other countries [12], [13]. Our univariate analyses showed the relationships of eleven potential confounding factors (gender, age, education, income level, occupation, family history of diabetes, region, BMI, cigarette smoking, alcohol drinking and exercise) with awareness, treatment and control of DM (Table 2). Multivariate logistic regression analyses showed the relationships between gender, age, education, income level, occupation, family history of diabetes, cigarette smoking, alcohol drinking and exercise with awareness, treatment and control of DM, as well as the relationships between age, income level, occupation, family history of diabetes, alcohol drinking and exercise with treatment of DM (Table 3). Prior work [13], [14] found that women were more likely than men to be aware of their risks of diabetes, and take their anti-diabetic medications, and those differences were also significant in this study. We also observed that the awareness and treatment of DM increased concomitantly with age, which is consistent with previous studies [15], [16]. This may be partly due to the relatively long course of diabetes, as it gives older people more time to accumulate diabetes knowledge. Moreover, life is relatively more stable for older people after retirement, giving them more energy and time to focus on the disease awareness, prevention and control. Previous studies had shown that high family monthly income may be associated with high awareness and treatment rates of DM [17], [18], but our study did not find these associations. The reason for this discrepancy needs further investigation. In addition, we found that participants with family history of diabetes were more likely than other members of the diabetes group to be aware of their blood glucose levels and take their anti-diabetic medications. However, cigarette smokers and alcohol drinkers were more likely to be unaware of their risks of diabetes, and this is consistent with prior studies [14], [19]. Our results suggest that cigarette smokers and alcohol drinkers should pay more attention to their blood glucose levels. The results of study also showed a pronounced relationship between frequent exercise and awareness and treatment of DM. Because we used a cross-sectional design in this our study, our ability to draw causal inference is limited. Therefore, we were unable to explain whether people who exercise more frequently will be more concerned about their blood glucose level or those who are aware of the diabetes condition will take anti-diabetic medications and exercise more frequently. However, it is certain that exercise is a behavioral intervention that DM patients usually undertake to control their high blood glucose levels [20], [21].

Although the univariate analyses of diabetes control showed that control among treated diabetes was associated with age, occupation and family history of diabetes, the multivariate analyses model only observed the relationship between mental labor, family history and diabetes control. Our study did not show statistically significant gender differences related to controlling glycemia. This finding agrees with other studies [22], [23], [24]. Importantly, we found that a family history of diabetes was associated with poor glycemic control and high diabetes treatment, and this is consistent with a prior study [25]; therefore, there is a need for increased public concern and regular surveillance for those who have a family history of diabetes.

In our study, the large sample size and precise physical measurements improved the validity of our results. Furthermore, the study contributed to meaningful insights concerning the current situation and associated risk factors of awareness, treatment and control of DM in northeast China. However, there are some limitations of this present study that should be mentioned. First, the awareness of diabetes was based on self-reported diagnosed DM. Second, the cross-sectional design might limit the ability to draw causal inference between the risk factors and DM. Third, the results in our study were from Jilin province, which may limit our ability to generalize the results to the whole of northeast China.

In summary, we observed that awareness and treatment rates of DM increased with age and higher in participants with a family history of diabetes and those who exercises frequently. The low control rate of diabetes among treated individuals was related to family history of the disease, which contributes to a high incidence of diabetes. Our results suggest that attempts to prevent diabetes should focus on the factors that lead to neglect of heath status; and effective preventive strategies should concentrate on young people and those who have a family history of diabetes, and include decreasing or cessation of alcohol consumption, smoking cessation or doing more exercise.
